# Diazoxide Promotes Oligodendrocyte Precursor Cell Proliferation and Myelination

**DOI:** 10.1371/journal.pone.0010906

**Published:** 2010-05-28

**Authors:** Birgit Fogal, Carolyn McClaskey, Sha Yan, Henglin Yan, Scott A. Rivkees

**Affiliations:** Department of Pediatrics, Section of Developmental Biology and Endocrinology, Yale Child Health Research Center, Yale University School of Medicine, New Haven, Connecticut, United States of America; Case Western Reserve University, United States of America

## Abstract

**Background:**

Several clinical conditions are associated with white matter injury, including periventricular white matter injury (PWMI), which is a form of brain injury sustained by preterm infants. It has been suggested that white matter injury in this condition is due to altered oligodendrocyte (OL) development or death, resulting in OL loss and hypomyelination. At present drugs are not available that stimulate OL proliferation and promote myelination. Evidence suggests that depolarizing stimuli reduces OL proliferation and differentiation, whereas agents that hyperpolarize OLs stimulate OL proliferation and differentiation. Considering that the drug diazoxide activates K_ATP_ channels to hyperpolarize cells, we tested if this compound could influence OL proliferation and myelination.

**Methodology/Findings:**

Studies were performed using rat oligodendrocyte precursor cell (OPC) cultures, cerebellar slice cultures, and an *in vivo* model of PWMI in which newborn mice were exposed to chronic sublethal hypoxia (10% O_2_). We found that K_ATP_ channel components Kir 6.1 and 6.2 and SUR2 were expressed in oligodendrocytes. Additionally, diazoxide potently stimulated OPC proliferation, as did other K_ATP_ activators. Diazoxide also stimulated myelination in cerebellar slice cultures. We also found that diazoxide prevented hypomyelination and ventriculomegaly following chronic sublethal hypoxia.

**Conclusions:**

These results identify KATP channel components in OLs and show that diazoxide can stimulate OL proliferation *in vitro*. Importantly we find that diazoxide can promote myelination *in vivo* and prevent hypoxia-induced PWMI.

## Introduction

In the United States, about 12% of infants are born prematurely [1], with very low birth weight (VLBW) (<1.500 gm) and extremely low birth weight (ELBW) (<1000 gm) infants accounting for about 20% of preterm births [1]. It is estimated that at least 25% of VLBL and ELBW infants will develop periventricular white matter injury (PWMI), which is one of the most common form of brain injury affecting premature infants [2] [3], [4]. PWMI can include either diffuse white matter disease or focal necrosis, consisting of periventricular lesions [4], [5], [6]. Diffuse PWMI appears to be far more common than focal necrosis [2] [3], [4].

PWMI is associated with significant morbidity, as affected individuals may have profound intellectual impairment and cerebral palsy [5], [7]. Highlighting the magnitude of PWMI, each year in the United States more than 400,000 infants are born prematurely [8]. Of these infants, about 100,000 are born at risk for PWMI, and about 25,000 children per year will develop PWMI. Finding a treatment for PWMI is thus of major clinical importance.

Oligodendrocytes (OLs) are the myelinating cells of the central nervous system [9], [10]. OL development to mature myelin forming cells follows a complex series of events during which progenitor cells undergo dramatic morphological and biochemical changes [11]. Four stages of OL differentiation are distinguished: oligodendrocyte precursor cells (OPCs), late OL progenitors, immature OLs, and mature OLs [11]. It is believed that loss of the proliferative OPCs plays a major role in PWMI causation [5]. Recently, we observed that hypoxia induces premature maturation of OPCs, leading to decreases in numbers of replicating OPCs, resulting in fewer myelinating OLs in the brain [12]. As such, premature OPC maturation may contribute to hypomyelination in the developing brain [12]. At present, pharmacological approaches that promote OPC proliferation leading to enhanced brain myelination are not clinically available.

Available evidence shows that OLs express ion channels, [13], [14], [15], [16] and changes in membrane potential and intracellular calcium levels influence OL development [17]. K channel blockers and depolarizing agents have been shown to cause G_1_ arrest in the OPC cell cycle [18]. Agents that hyperpolarize OLs promote OL proliferation [14], [15].

Compounds that influence K-channel activity are currently available for clinical use, and include diazoxide, which activates K_ATP_ channels [19], [20]. K_ATP_ channels require two structurally diverse subunits [19], [20]. One subunit is a member of the pore-forming inward rectifier Kir6.x family of potassium channels, while the other subunit is a sulfonylurea receptor (SUR) [19], [20], that belongs to the ATP-binding cassette superfamily.

We are unaware of studies that have examined the expression of Kir6 or SUR proteins in OLs. It is also not known if K_ATP_ channel antagonists or agonists effect OL development or function. We postulated that alteration in K_ATP_ channel activity could have potential utility as a therapeutic agent in white mater injury. To test this hypothesis we studied cultured OLs and validated models of PWMI [21].

## Materials and Methods

### Animals

This study was conducted in accordance with USDA guidelines for the use of experimental animals and was approved by the Institutional Animal Care and Use Committee (IACUC) of Yale University School of Medicine. CD1 and C57BL/6 mice and Sprague Dawley rats were obtained from Charles River Laboratories (Wilmington, MA). The Yale IACUC approval number for this study was #2008-11198. The Yale University Public Health Services approval number is A3230-01.

### Cell culture

Purified OPC cultures were prepared as described [22], [23]. In brief, primary rat mixed glial cell cultures were isolated from whole brains of postnatal day (P) 1 rats, dissociated into single cells, and cultured into poly-D-lysine (PDL, Sigma-Aldrich, St. Louis, MO) coated T75 tissue culture flasks. Plating medium consisted of Dulbecco's modified Eagle's medium (DMEM, Invitrogen, Carlsbad, CA) supplemented with 10% fetal bovine serum (FBS; InVitrogen, Carlsbad, CA), 2 mM L-glutamine, 100 µM streptomycin, and 10 µg/ml penicillin. Tissue cultures were maintained at 37°C in a humidified 5% CO_2_ incubator, and medium was exchanged every 3 days. Once confluent (after 7–9 days), microglia were separated by mechanical shaking of flasks on a rotary shaker for 60 min at 200 rpm and removed. After addition of fresh medium, the remaining cells were allowed to recover overnight before repeating the mechanical shaking for an additional 16 h at 200 rpm to isolate OPCs. To ensure purity of OPC cultures, the isolated cells were transferred to a tissue culture dish, from which the loosely attached OPCs were detached by gentle shaking after 60 min, leaving behind attached microglia and astrocytes. OPCs were plated onto PDL coated 96 well plates using an automated dispenser and allowed to adhere to the plates over the next 1–2 days. In agreement with others [24], this procedure yields 98% A2B5-positive (OPC marker), and 2% MBP-positive (mature OL marker) cells. GFAP-positive (astrocyte marker) or Ox2A-positive (microglia marker) cells cannot be detected in cultures prepared in this manner.

### Drug exposure

Cells were exposed to K_ATP_ channel stimulators for 72–96 h. The compounds included commercially available K_ATP_ channel activators ZM26600 (2.5 uM), Pinacidil (10 uM), Y26763 (200 nM), Levcromakalim (2.5 uM), P1075 (100 nM) and diazoxide. Cultures were washed two times with DMEM to remove any residual serum and then the respective solutions of K_ATP_ channel activators were added daily in an incubation buffer of DMEM with 0.5% FBS +10 µg/ml biotin and N2 supplement. Cells were then placed at 37°C in a humidified atmosphere containing 5% CO_2_.

### Quantification of cell number

To evaluate cell proliferation in response to K_ATP_ channel stimulators, we utilized the CyQUANT® NF Cell Proliferation assay (Invitrogen, Carlsbad, CA) according to the manufacturer's instructions. This assay measures cellular DNA content as a direct index of cell proliferation. Since cellular DNA content is highly proportional to cell number, this is a very accurate way to assess cell proliferation specifically [25]. At the end of drug exposure, medium was removed and a stock solution of the green-fluorescent CyQUANT GR dye (prepared according to manufacturer's instructions) was added. Upon binding to DNA, the GR dye shows a measurable enhancement in fluorescent intensity. Cells were returned to the incubator (37°C) for 2 h, which we determined to result in maximal and stable changes in fluorescence. Fluorescence was measured using an Envision Multilabel reader (Perkin-Elmer; Excitation: 480 nm, Emission: 530 nm).

### Calcium imaging

Calcium imaging was performed by using an Olympus Fluoview confocal laser scanning microscope (IX70, Melville, NY), an UPlanFl 20× objective (N.A. 0.5), and Fluoview image processing software (v2.1). To assess intracellular calcium levels the calcium ionophore Fluo-3 was used.

Cells were first evaluated before the addition of any drug. After addition of drug, calcium levels were recorded. Quantification and statistical analysis of quantified data was performed by using Fluoview software, Microsoft Excel 2000 (Microsoft, Inc., Redmond, WA), and GraphPad Prism (v3.0, GraphPad Software, Inc., San Diego, CA), as described [26].

### Cerebellar Slice Cultures

Cerebellar organotypic cultures of mice (postnatal 0 day) were prepared as described [27], [28]. 300-µm thick slices were transferred onto membranes of 30 mm Millipore culture inserts with a 0.4 µm pore size, and maintained in six-well tissue culture plates containing 1 ml of medium at 35°C in room air with 5% CO_2_. The nutrient medium consisted of 50% basal medium with Earle's salts, 25% horse serum, 25% Hank's balance solution, 1 mM L-glutamine, and 5 mg/ml glucose. After 24 h the medium was removed and culture plates were washed twice with phosphate-buffered saline before addition of tolbutamide, diazoxide, or vehicle in N1 medium. After five days cultures were immunostained for MBP as described below. Total numbers of MBP-positive cells and myelinated fibers per mm^2^ area were determined for each slice by manual counting by an individual who was blinded to treatment conditions. Each treatment condition contained six slices from three different animals.

### Polymerase chain reaction

cDNA was made from OPCs and mature OLs. DNA was amplified from cells and used in PCR reactions with SUR or KIR6 subtype-specific primers, as detailed [29].

### Western blot analysis

Cells were washed in ice-cold phosphate buffered saline (PBS) and lysed using hot lysis buffer containing 42 mM Tris-HCl (pH 6.8), 1.3% sodium dodecylsulfate (SDS), 6.5% glycerol, and 0.1 mM sodium orthovanadate. Protein concentrations were determined using the bicinchonic acid method (BCA kit, Pierce Technologies, Rockford, IL). Before loading, samples were mixed with 10 mM dithiothreitol and 0.1% bromophenol blue, and boiled for 5 min. Proteins were separated on an SDS-polyacrylamide gel, transferred to a polyvinylidene fluoride (PVDF) membrane, and blocked with 5% non-fat dry milk in 20 mM Tris-HCl, pH 7.6, 150 mM sodium chloride and 0.1% tween-20 (TBS-T) for 1 hr. Anti Kir6.1 and 6.2 and SUR1 and 2a antisera were obtained from Santa Cruz Biotechnology (Carlsbad, CA). Secondary antibodies were diluted in blocking buffer and incubated for 1 h at RT. Horseradish peroxidase-conjugated goat anti-rabbit antibody (1∶5000 dilution) was used to detect labeled product. After washing, proteins were detected using an enhanced chemiluminescence kit (Pierce Biotech).

### *In vivo* Hypoxia

C57BL/6 mice were exposed to low or normal oxygen conditions from P3-P12 as described [21], [30]. In brief, litters of pups (P3) were placed with the dam in a Plexiglas chamber, in which oxygen levels were maintained at 9.5±1.0%., O_2_ levels were continuously monitored using a Cameron Instrument (Ontario, Canada) dual channel oxygen monitor attached to O_2_ electrodes placed at each end of the chamber. Control animals were kept in room air (∼22%) outside the Plexiglas chamber. Because C57BL/6 dams do not normally care for pups in hypoxic conditions, pups (hypoxia and control) were cross-fostered with CD1 dams, which were placed in the same cage before birth of pups. Animals were removed from the chamber daily for less than 15 min to allow for diazoxide (10 mg/kg i.p.) or vehicle administration, as well as observation of weight gain. Mice were euthanized at P12, brains were harvested and shock frozen in ice cold (−20°C) 2-methylbutane and stored at −80°C until assessment of ventricular size or MBP immunocytochemistry (see below). At least eight animals were studied in each treatment group. There were no difference in mortality observed between the vehicle and diazoxide treated pups. Mortality rates were less than 12% for each treatment group (p>0.05).

### Quantification of ventricular size

Ventricle size was determined as reported [30], [31]. Animals were weighed, anesthetized, and decapitated. Brains were shock frozen in ice cold (−20°C) 2-methylbutane and stored at −80°C. Coronal sections spanning the brain were cut in a cryostat at a thickness of 16 µm in a Zeiss cryostat. Sections were mounted onto glass slides and stained with Phoenix Blue (Thermo Scientific, Waltham, MA). Serial sections through the midstriatum were photographed to include the lateral ventricle region. Ventricular sizes were quantified using Sigma Scan Pro Image Analysis Version 5.0.0 (SPSS Inc., Chicago, IL). The ventricular area, outlined by the Phoenix Blue staining, was measured and numerically integrated across the thickness of the slice. Images were obtained using a Leica florescence microscope.

### Immunostaining

Tissue sections were fixed with 4% paraformaldehyde in PBS for 30 min at room temperature and incubated with 10% normal goat serum plus 0.3% Triton-X-100 in 0.1 M PBS (pH 7.4) overnight to block nonspecific binding. The sections were incubated with monoclonal MBP antibody (SMI-99, Covance, Princeton, NJ) at a dilution of 1∶1000 in PBS with 10% normal goat serum plus 0.1% Triton-X-100 at 4°C overnight, followed by incubation of Alexa Fluor 495 anti-mouse IgG (Molecular Probes) at a dilution of 1∶400. The intensity of labeling was assessed using Image J Version 1.42q (National Institutes of Health, Bethesda MD) at the mid-level of the corpus callosum from three slides per animal.

For A2B5 and O1, immunocytochemistry was performed as described [32]. Cultures were washed twice with L-15 medium. Cells were then incubated with murine monoclonal antibodies, or A2B5, O1 hybridoma culture supernatants for 20 min at 37°C. Cells were incubated with Alexa Fluor 594- or Alexa Fluor 488-conjugated antimouse secondary antibody (1∶200) for 20 min at 37°C.

For Kir and SUR immunostaining, cells were fixed with 4% paraformaldehyde and treated with 0.2% Triton X-100 and 10% goat serum. Next, cells were incubated with monoclonal anti-Kir6.1 and 6.2 and SUR1 and 2a (Santa Cruz Biotechnology; Carlsbad, CA) (1∶100-500) or mouse IgG (control) plus 10% normal goat serum overnight at 4°C. After washing three times with PBS, cells were incubated with goat anti-mouse IgG conjugated to Alexa Fluor 594, or goat anti-rabbit IgG conjugated to Alexa Fluor 488 for 90 min at room temperature. Cells were examined by fluorescence microscopy.

### Statistical Analysis

All experiments were repeated at least three times and statistical analyses were performed using Graph Pad Prism software. Comparisons among multiple groups were made by ANOVA, with Bonferonni post-test comparisons. Paired comparisons were made by the Students-T test. In all experiments significance was assessed at p<0.05.

## Results

### Expression of SUR and KIR6 in OLs

We postulated that OLs contain K_ATP_ channels that could be pharmacological targets. To begin to address this issue, we examined the expression K_ATP_ channel components SUR and KIR6.1 and 6.2 in OLs. First, we assessed KIR6.1 and 6.2 and SUR gene expression in OLs by PCR. cDNA was made from OPCs and mature OLs. For OPC studies, 98% of cells were A2B5-postive; for mature OL studies, 95% of cells were MBP-positive.

**Figure 1:**
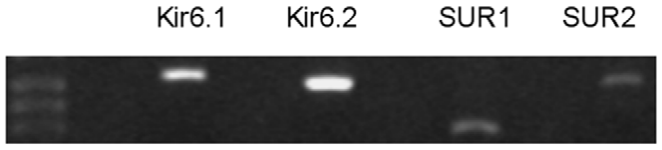
PreOLs express KIR6.1, KIR6.2, SUR1 and SUR2 genes. cDNA was prepared from OPCs and used in PCR reactions with primers specific to each gene. 98% of cells were A2B5-postive indicating that cells were OPCs. Left, molecular weight markers. White bands show amplified products. Data shown are representative of three separate studies performed on OPC cultures prepared at different times. Similar observations (not shown) were seen for mature OLs (95% MBP-positive).

DNA was amplified from cells and used in PCR reactions with SUR or KIR6 subtype-specific primers, as reported [29]. KIR6.1 and KIR6.2, as well as SUR1 and SUR2 mRNA expression was observed in both, OPCs (Fig. 1) and mature OLs (data not shown).

**Figure 2:**
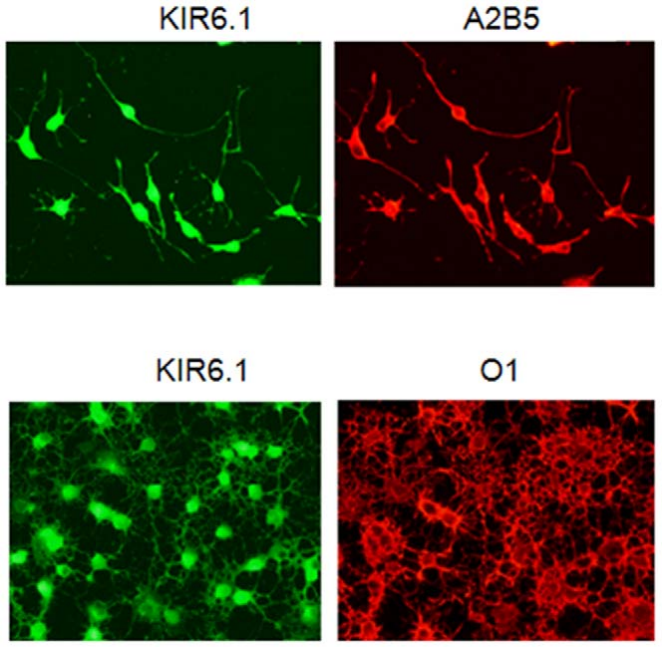
OPCs express KIR6.1. OLs derived from neonatal rat brain were isolated and cultured. Double-labeling immunostaining shows staining for KIR6.1 in either A2B5 or O1-positive OLs. Data shown are representative of three separate studies performed on OPC cultures prepared at different times.

**Figure 3:**
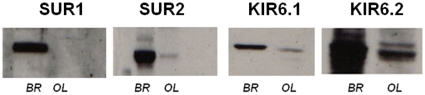
OPCs express SUR and KIR protein. OPCs derived from neonatal rat brain were isolated and cultured. Western blotting was performed on whole brain lysates and OPCs. Approximate sizes of bands were SUR1, 140 kDa; SUR2, 180 kDa; Kir6.1 70 kDa; Kir6.2, 60 kDa. OL, oligodendrocyte cultures; Br, whole brain. Data shown are representations of three separate studies using OPCs prepared at different times.

Next, we examined SUR and Kir protein expression using immunoblotting. We observed bands of the appropriate sizes for Kir6.1 (51 kDa), Kir6.2 (40 kDa), SUR1 (150 kDa), and SUR2 (180 kDa) in protein lysates prepared from whole brain. Using lysates from OPCs, Kir 6.1, Kir6.2, and SUR2, but not SUR1 protein expression was detected (Figure 2). These results were confirmed by immunocytochemistry, in which Kir6.1, Kir 6.2, and SUR2 positive cells were detected in both OPC (A2B5 positive) and mature OL (O1 positive) cultures, whereas, SUR1 labeling was not present (Figure 3). These data show that OPCs express SUR2 and KIR6.1 and 6.2 genes and proteins, which are components of K_ATP_ channels.

### Influences of K_ATP_ activation on OPC proliferation

We next assessed the potential of K_ATP_ activation to stimulate OPC proliferation using the K_ATP_ channel activator diazoxide [33]. Cultured OPCs were treated with concentrations of diazoxide ranging from 0.1 to 100 µM for 72–96 h. To evaluate cell proliferation in response to K_ATP_ channel stimulators, we utilized the CyQUANT® NF Cell Proliferation assay (Invitrogen, Carlsbad, CA) which is a validated method fro assessing cell proliferation [25]. This assay measures cellular DNA content as a direct index of cell proliferation. Since cellular DNA content is highly proportional to cell number, this is a very accurate way to assess cell proliferation specifically [25].

**Figure 4:**
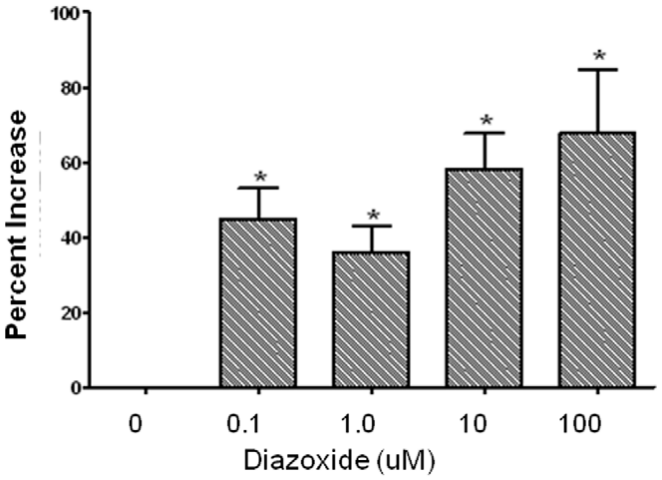
Concentration-response effects of diazoxide on PreOL proliferation. Data shown are from three studies in which each concentration was tested in triplicate in each study. * p<0.05, vs. vehicle, ANOVA. Mean ± SEM shown. Y-axis represents the percent increase relative to control in cellular DNA content, which is a direct index of cell proliferation.

**Figure 5:**
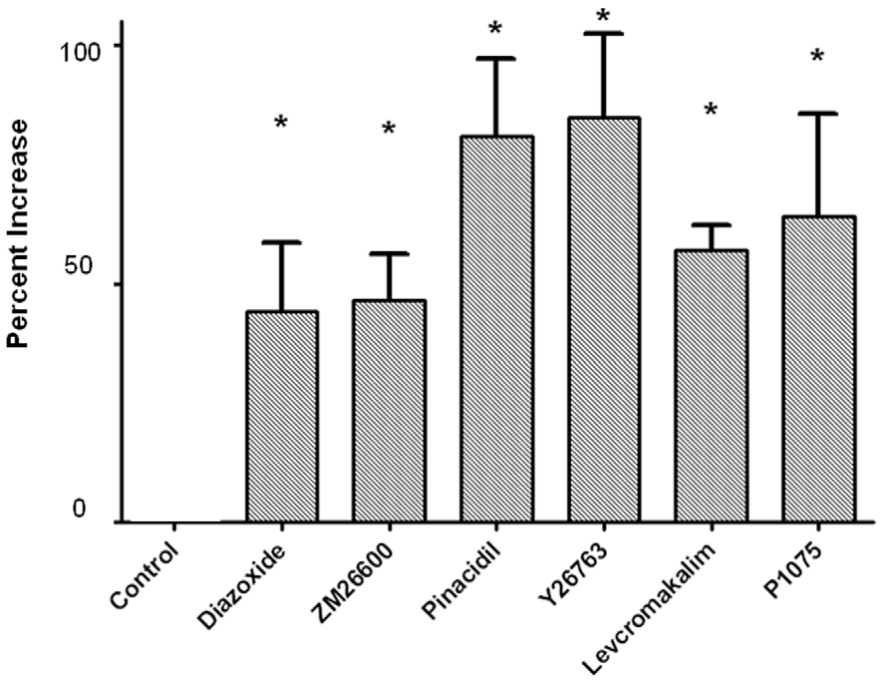
Effects of known K_ATP_ channel activators on OPC proliferation. Diazoxide (10 uM), ZM26600 (2.5 uM), Pinacidil (10 uM), Y26763 (200 nM), Levcromakalim (2.5 uM), P1075 (100 nM). n = .5-10 (from two separate experiments). Data shown are from three studies in which each concentration was tested in triplicate in each study. * p< 0.05, vs. vehicle, ANOVA. Mean ± SEM shown. Y-axis represents the percent increase relative to control in cellular DNA content, which is a direct index of cell proliferation.

Diazoxide stimulated OPC proliferation in a dose-dependent manner (Fig. 4). Interestingly, a typical concentration-response curve was not observed suggesting that a threshold level of drug may need to be achieved to trigger a response. We also tested the effect of other commercially available K_ATP_ channel activators on OPC proliferation. Namely, ZM26600 (2.5 uM), Pinacidil (10 uM), Y26763 (200 nM), Levcromakalim (2.5 uM), P1075 (100 nM) were tested using fixed doses, based on known effective concentrations [34]. As with diazoxide, OPC proliferation was increased by these known K_ATP_ channel activators, with several compounds being more potent than diazoxide (Fig. 5).

### Influences on intracellular calcium levels

We next examined if K_ATP_ activators and blockers influence intracellular calcium levels [35], as changes in K_ATP_ activity influence intracellular calcium levels and cell proliferation [35]. After OPCs were loaded with the calcium ionophore Fluo3/AM, they were treated with diazoxide (10 uM) while being visualized by confocal imaging. We also tested the K_ATP_ channel blocker tolbutamide (100 uM). Tolbutamide blocks K_ATP_ channels, thus we tested if this compound influence intracellular calcium levels in a way opposite that observed for diazoxide which activates K_ATP_ channels.

**Figure 6:**
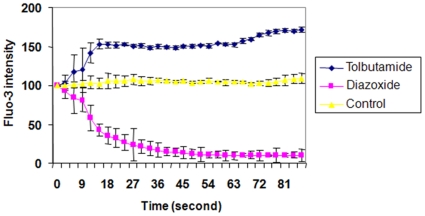
Diazoxide inhibits intracellular calcium accumulation. Data points are the mean ± SEM of 30 separate cells. 100 depicts baseline Fluo-3 intensity. Drugs administered at 0 seconds. These data are representative of 3 separate studies. Tolbutamide (100 uM), diazoxide (10 uM).

We observed that intracellular calcium levels decreased when cells were treated with diazoxide. In contrast, addition of tolbutamide to the medium induced prompt intracellular calcium increases (Fig. 6).

### Influences on myelination *in vitro*

After the above studies, we assessed if K_ATP_ channel activation or blockade influences myelination using cerebellar slice cultures. In cerebellar brain slices, microglia, OLs and neurons are present [28]. In addition, axon growth and myelination occurs in slices, providing an excellent model for examining influences on myelination and axon growth [36].

Slices were prepared from P0 mice. After 2 days in culture, slices were treated with diazoxide (10 uM) or tolbutamide (100 uM) for 5 days, followed by immunostaining for MBP. As above, because tolbutamide blocks K_ATP_ channel activation, we tested if this compound influence slice culture myelination in a way opposite that observed for diazoxide.

**Figure 7:**
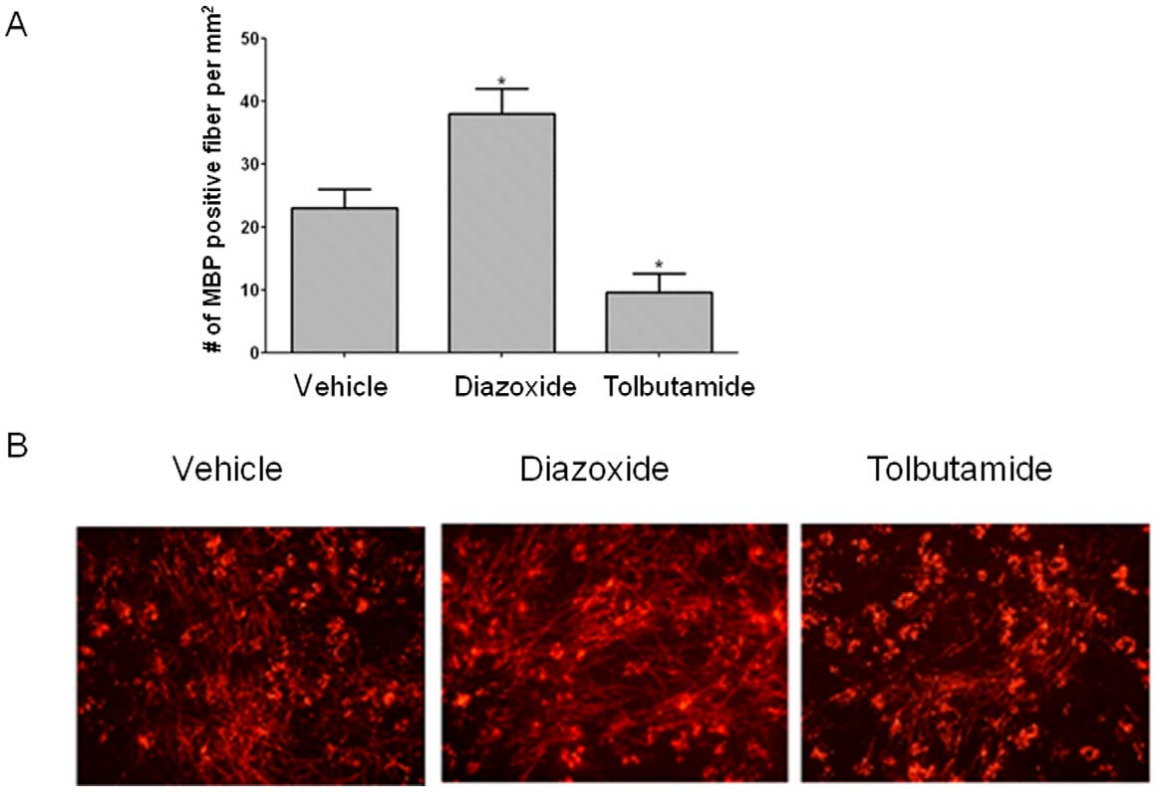
Diazoxide stimulates myelinated fiber formation. Slice cultures from P0 cerebellum were treated with diazoxide (1 uM), tolbutamide (100 uM), or vehicle for 5 days. Slices were stained for MBP. A. Top panel shows quantitative assessment of myelinated fiber number (p<0.01; ANOVA). Data are mean ± SEM from six separate slices per treatment. B. Images of MBP labeled specimens.

In the diazoxide-treated slices, we observed large numbers of myelinated fibers (38±4 MBP-positive fibers/slice) that exceeded numbers observed with vehicle treatment (23±3 MBP-positive fibers/slice) (Fig. 7). In tolbutamide-treated specimens, myelinated fibers were sparse (9.6±3 MBP-positive fibers/slice) and less than that observed in vehicle-treated slices.

### Protective effects of diazoxide on hypoxia-induced PWMI

Considering the effects of diazoxide treatment on OPCs and myelination observed *in vitro*, we assessed if diazoxide could confer protection against hypoxia-induced PWMI using the chronic sublethal hypoxia model [21], [30]. Whereas different models have been used to recapitulate human infant white matter injury [37], no model exactly recapitulate the human lesion. However, the chronic sublethal hypoxia model mirrors the diffuse PWMI phenotype [21], which is the most common form of white matter injury in premature infants.

C57BL/6 mice were reared in room air or 10% O_2_ from P3 to P12, and treated daily with diazoxide (10 mg/kg i.p.) or vehicle. The dose of 10 mg/kg was used as this is typically used in clinical settings [38].

We observed that mice reared under hypoxic conditions gained less weight than mice reared in normoxia. Weight gain overtime was similar in mice treated with vehicle or diazoxide under normal oxygen conditions. However, diazoxide-treated animals show a trend for better weight gain under hypoxic conditions, although this difference was not significant.

**Figure 8:**
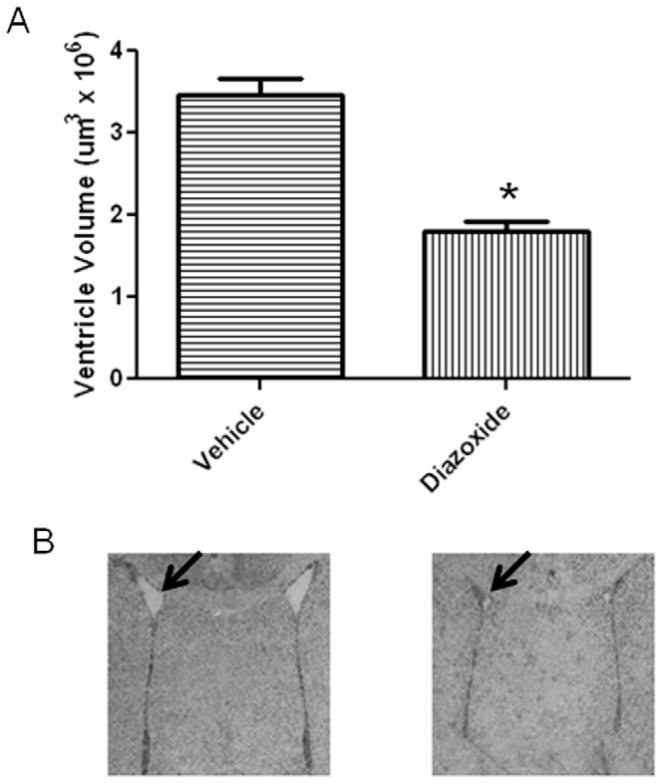
Animals reared in chronic hypoxia and treated with diazoxide demonstrate reductions in ventriculomegaly. A. Top panel shows quantitative assessment of ventricle size. Data shown are mean ± SEM from one experiment with 4–6 animals per treatment group (p<0.01; t-test). Similar results were obtained in another separate study performed at a different time. Chronic hypoxia caused pronounced ventriculomegaly (arrow). Note the reduction in ventricle size in the mice treated with diazoxide. B. Photographs are from one animal in each treatment group. * p<0.01 ANOVA.

When ventricle size was assessed (Fig. 8), we observed ventriculomegaly in the hypoxia-vehicle group (2.86×10^6^ µm^3^±0.3×10^6^). In the hypoxia-diazoxide group, the ventricles were not enlarged (1.37×10^6^ µm^3^±0.2×10^6^) (p<0.05; ANOVA). In normoxic conditions ventricle sizes were similar in pups treated with vehicle (1.93×10^6^ µm^3^±0.6×10^6^) or diazoxide ((1.58×10^6^ µm^3^±0.4×10^6^) (p>0.05; ANOVA).

**Figure 9:**
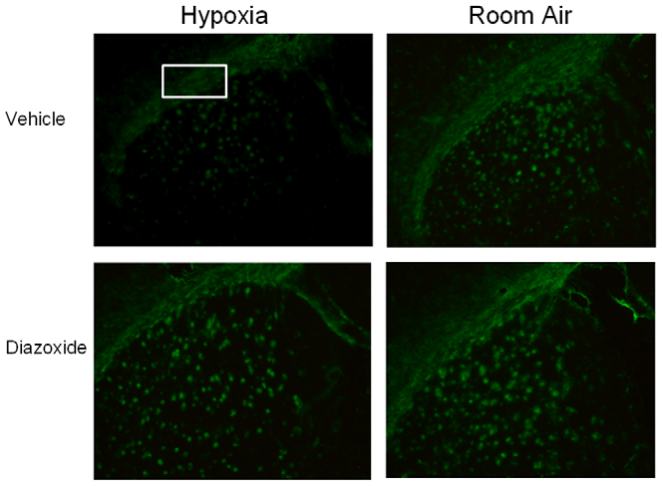
Animals reared in chronic hypoxia and room air demonstrate increased myelination with diazoxide treatment. Coronal images at level of corpus callosum shown are from one experiment with 4–6 animals per treatment group and are representative of one other separate study performed at a different time. MBP staining was performed at the same time. Box depict region of corpus callosum where labeling intensity was assessed. Photographs were taken at identical exposures. Hypoxia caused diffuse reduction in cerebral MBP-labeling, which was markedly improved with diazoxide. We also observed more MBP-labeling in diazoxide-treatment mice reared in room air compared to those treated with vehicle.

**Figure 10:**
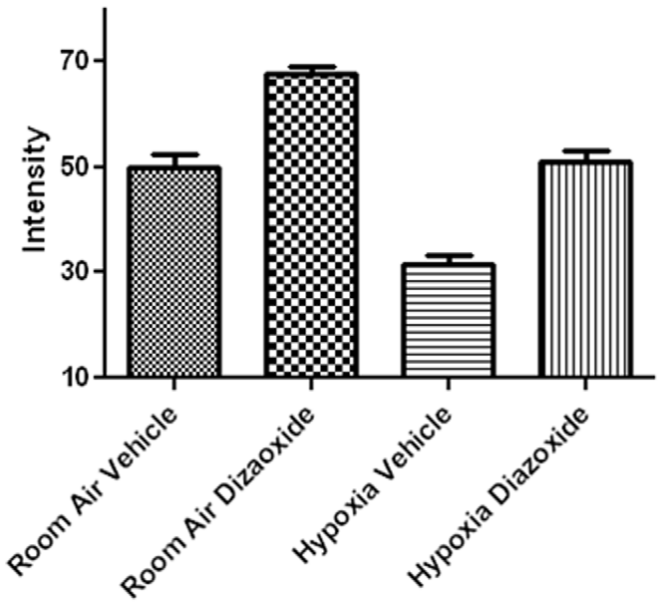
Animals reared in chronic hypoxia and room air demonstrate increased myelination with diazoxide treatment. Quantitative assessment of labeling using Image J Version 1.42q (National Institutes of Health, Bethesda MD) at the mid-level of the corpus callosum of groups shown in Fig. 9. Data shown are mean ± SEM from one experiment with 3–6 animals per treatment group. Similar results were obtained in another separate study performed at a different time.

Additionally, myelination was assessed via immunostaining for MBP. Compared to animals reared under normal oxygen conditions, animals kept under hypoxia showed a reduction in MBP labeling. Importantly, in diazoxide-treated mice reared under hypoxia, MBP labeling appeared similar to control animals. (p<0.01, ANOVA; Figs. 9 and 10). Interestingly, diazoxide treatment in animals reared under normoxic conditions manifested slight, but significant increases in MBP staining (p<0.01, ANOVA; Figs. 9 and 10).

## Discussion

The above data identify diazoxide as a potent stimulator of OPC proliferation and myelination. We observe that OPCs express K_ATP_ channels and that diazoxide and other K_ATP_ channel activators stimulate OPC proliferation. We also show that diazoxide can prevent hypoxia-induced ventriculomegaly and hypomyelination, which are features of PWMI.

Available evidence suggests that it is possible to alter OPC proliferation and differentiation with exogenous compounds including platelet-derived growth factor (PDGF) and basic fibroblast growth factor (bFGF) [39]. None of these compounds, though, are available for clinical use, and their potential therapeutic utility is limited by their peptide nature [39].

We find that diazoxide, which activates K_ATP_ channels [33], stimulates OL proliferation and promotes myelination during early development. Diazoxide acts on inwardly rectifying K_ATP_ channels that close when ATP levels are high [33], [35]. Other K_ATP_ channel activators include ZM26600, pinacidil, Y26763, levcromakalim, and P1075 [33]. We observed that the other K_ATP_ channel activators tested stimulated OPC proliferation, as well.

K_ATP_ channels are unique among Kir channels in that they require two structurally diverse subunits in a stoichiometry of 4∶4 to form functional channels [20]. One subunit is a member of the inward rectifier Kir6.0 family of potassium channels; the other subunit is a sulfonylurea receptor (SUR) [19], [20]. The subunit composition of K_ATP_ channels varies among different tissues, with different SUR subtypes interacting with Kir6.1 or Kir6.2 [19], [20]. Kir6.2/SUR1 is found in pancreatic cells; Kir6.2/SUR2A is found in cardiac K_ATP_. Kir6.1/SUR1 is found in mitochondrial channels [40], [41]. We observed that OL expressed both Kir6.1 and Kir6.2 and SUR2. Although we observed SUR1 gene expression by PCR, we did not detect protein expression either by immunoblotting or immunocytochemistry.

Interestingly, the sensitivity of K_ATP_ channels to the different K_ATP_ channel activators depends in large part on their subunit composition [34]. For example, pancreatic K_ATP_ channels composed of SUR1/Kir6.2 subunits are highly sensitive to diazoxide, but less sensitive to pinacidil and levcromakalim, while cardiac K_ATP_ channels (SUR2A/Kir6.2) are activate by cromakalim and pinacidil but not diazoxide [34]. However, diazoxide can also activate Kir6.1 alone [42]. Although beyond the scope of this study to determine the exact composition and stoichiometry of OL K_ATP_ channels, the observed effects of the different K_ATP_ channel activators on OPC proliferation supports the presence of SUR2A/B and Kir6.1/Kir6.2, subunits in OPCs.

Available evidence shows that OLs express ion channels [14], [15], [16]. Although not extensively studied, changes in membrane potential and intracellular calcium levels have been observed to influence OL development [17]. In rats, OLs express an inwardly rectifying K-current (IKIR), which are G protein-regulated [43]. Kir4.1 expression has been detected in OLs [44]. Showing that altered channel activity alters OL development, blockade of K channels in OPCs inhibits cell proliferation [45], [46]. Studies of Kir4.1 knockout mice reveal undermyelination of the brain [14], suggesting that the Kir4.1 channel subunit is crucial for OL maturation.

K channel blockers and depolarizing agents cause G_1_ arrest in the OPC cell cycle. There is also accumulation of p27^Kip1^ and p21^CIP1^ in OLs, which regulate cell proliferation and differentiation [47], [48], [49], [50]. Elevated p27 is associated with premature exit from the cell cycle and cessation of proliferation [47], [48], [49], [50]. These effects on cell proliferation appear to involve changes in intracellular calcium levels [17], [51], [52]. When intracellular calcium levels rise, proliferation is reduced in favor of maturation [51], [52]. When intracellular calcium levels fall, proliferation is increased and cells do not mature [51], [52]. Consistent with this notion, we find that diazoxide results in decreases in intracellular calcium levels, whereas tolbutamide triggers increases in intracellular calcium levels. It is important to highlight that we only examined acute changes in intracellular calcium levels in response to treatment with diazoxide and tolbutamide. Future studies are indicated to discern the duration of such responses and whether chronic exposure to if K_ATP_ channel agonists and antagonist influences changes in cell membrane potential and intracellular calcium levels.

When we examined myelination in slice culture models, we observed that diazoxide stimulated myelination and tolbutamide inhibited myelination. Interestingly, in newborn mice treated with diazoxide in room air, we observed that diazoxide stimulated myelination, above that observed in control animals. Further studies are thus indicated to assess if K_ATP_ channel agonists could result in hypermyelination or abnormal dysmyelination.

It is also important to note that the cerebellar slice culture model and the *in vivo* studies of developing pups provided complementary models for assessing effects of diazoxide on myelination. In both models, we observed that there was more myelination in the diazoxide-treated groups than those treated with vehicle. The presence of increased myelination in the two different model systems supports the notion that diazoxide can indeed promote myelination in different brain regions.

We recognize that no in vivo model completely recapitulates human white matter injury. Thus in future studies it will be interesting to examine effects in other models of white matter injury, such as those caused by hypoxic-ischemic or inflammatory insults. Whereas it is likely that the favorable effects of diazoxide on myelination in the models used represents are direct effects on OLs, additional studies are indicated to determine if this is a direct effect of diazoxide or an indirect effect mediated by other brain cell types. Studies of ultrastructural analysis will also be revealing in assess axon myelination.

Previously, we reported that caffeine confers protective effects against hypoxia-induced white matter injury in development [31]. In comparison with those observations, in the identical model, we find diazoxide to be more effective in promoting myelination. We also recently performed high-throughput screening of chemical libraries using the GenPlus Custom Collection (MicroSource Discovery Systems, Inc; Gaylordsville, CT) and a library from Maybridge (Cornwall, UK). Of 14,700 compounds screened, none were as potent as diazoxide in stimulating OPC proliferation under the same experimental conditions (SAR and BF, unpublished observations).

Currently, diazoxide is FDA approved for the treatment of hyperinsulinism in infants [53]. Diazoxide has been used in infants for extended periods with a favorable safety profile [54]. Considering that no FDA approved drugs are currently available for the treatment of PWMI or other hypo- or demyelinating conditions, further studies are indicated to assess the utility of diazoxide as a potential therapeutic. It is also possible that other K_ATP_ channel activators that specifically target OL K_ATP_ channel components may prove to be even more effective.
